# sFasL—The Key to a Riddle: Immune Responses in Aging Lung and Disease

**DOI:** 10.3390/ijms22042177

**Published:** 2021-02-22

**Authors:** Shulamit B. Wallach-Dayan, Dmytro Petukhov, Ronit Ahdut-HaCohen, Mark Richter-Dayan, Raphael Breuer

**Affiliations:** 1 Lung Cellular and Molecular Biology Laboratory, Institute of Pulmonary Medicine, Hadassah Medical Center, The Hebrew University of Jerusalem, Jerusalem 91120, Israel; petukhov@hadassah.org.il (D.P.); raffibreuer@gmail.com (R.B.); 2 Department of Medical Neurobiology, Institute of Medical Research, Hadassah Medical School, The Hebrew University of Jerusalem, Jerusalem 91120, Israel; ronit.ahdut@mail.huji.ac.il; 3 Department of Science, The David Yellin Academic College of Education, Jerusalem 9103501, Israel; 4 Department of Emergency Medicine, Hadassah Medical School, The Hebrew University of Jerusalem, Jerusalem 91120, Israel; richter@hadassah.org.il

**Keywords:** soluble Fas ligand, aging, autoimmune response, pulmonary disease

## Abstract

By dint of the aging population and further deepened with the Covid-19 pandemic, lung disease has turned out to be a major cause of worldwide morbidity and mortality. The condition is exacerbated when the immune system further attacks the healthy, rather than the diseased, tissue within the lung. Governed by unremittingly proliferating mesenchymal cells and increased collagen deposition, if inflammation persists, as frequently occurs in aging lungs, the tissue develops tumors and/or turns into scars (fibrosis), with limited regenerative capacity and organ failure. Fas ligand (FasL, a ligand of the Fas cell death receptor) is a key factor in the regulation of these processes. FasL is primarily found in two forms: full length (membrane, or mFasL) and cleaved (soluble, or sFasL). We and others found that T-cells expressing the mFasL retain autoimmune surveillance that controls mesenchymal, as well as tumor cell accumulation following an inflammatory response. However, mesenchymal cells from fibrotic lungs, tumor cells, or cells from immune-privileged sites, resist FasL^+^ T-cell-induced cell death. The mechanisms involved are a counterattack of immune cells by FasL, by releasing a soluble form of FasL that competes with the membrane version, and inhibits their cell death, promoting cell survival. This review focuses on understanding the previously unrecognized role of FasL, and in particular its soluble form, sFasL, in the serum of aged subjects, and its association with the evolution of lung disease, paving the way to new methods of diagnosis and treatment.

## 1. Introduction

Fas ligand (FasL, CD95L, CD178), a 40 kDa cell surface transmembrane protein, is known to be primarily expressed by activated T-cells [1] and their released extracellular vesicles, from prenatal to somatic stages [2,3,4]. It forms multimere complexes [5] to elicit cell death by induction of a signaling complex (DISC), which binds to Fas (CD95), a TNF-family cell death receptor [4]. The Fas death receptor is commonly expressed on so called “target” cells, such as lung epithelial cells, fibroblasts, T-lymphocytes, virally infected cells and tumor cells [1,3,6]. In some conditions, these same cells (tumors, fibroblasts from fibrotic sites) may also express the Fas ligand, and transform from a defensive situation to an offensive one by counterattacking cells of the immune system and escaping “immune surveillance” just as do cells in immune-privileged sites (e.g., testis Sertoli cells, eye corneal epithelium and neurons) [1,7]. Membrane FasL also enabled myofibroblasts to attain a cytotoxic phenotype and eliminate alveolar epithelial cells and T-cells [7,8].

A soluble form of FasL exists (sFasL), and is known to compete with the full, membrane-anchored protein (mFasL) on the binding site of the Fas death receptor [9], that not only joins this modulation of immune responses [1,2,5], but also prompts cell motility [2,10,11,12].

In this review, we will discuss new perceptions of mechanisms of sFasL increments, mostly in blood serum, and its role in lung health and disease during aging. 

This knowledge is important for understanding the role sFasL plays in various conditions of the aging and diseased lung, with emphasis on immunological disorders, including viral infection, and may pave the way for appropriate molecular diagnostics and therapies.

## 2. Serum Levels of Soluble FasL Increase with Age

Aging is a physiological process characterized by an “inflammaging” response, i.e., a low-grade inflammation comprising the senescence-associated secretory profile (SASP) [13] of pro-inflammatory and cell death effectors affecting tissue repair, regeneration and promoting premalignant responses [13]. Senescence is associated with a variety of lung diseases, including those mediated by viral infection [14,15]. An important SASP-associated cytokine is the Fas ligand. Membrane FasL expression, for example, in T-cells, was shown to decrease with age in the blood serum of healthy individuals [16], whereas the soluble form increased [17,18,19] (see Table 1). A similar increase in sFasL levels was detected in the serum of Werner syndrome patients, a typical accelerated aging syndrome, which was associated with chronic inflammation [17]. Although a few studies reported a mild decrease in the age-related increments of sFasL in serum in normal subjects [20,21], and they were even lower in athletes [22] (Table 1), its increments with aging were further detected in animal studies [5]. In particular, the percentage of activated rat T-lymphocytes expressing FasL increased from about 5% in cells from young animals to more than 50% in old counterparts [23]. This phenomenon and its mechanisms are further detailed.

## 3. Molecular Signaling and Mechanism of Soluble FasL Increment with Age

The mechanism behind soluble FasL increments in the serum of aging subjects has not been determined with certainty. 

**Figure 1:**
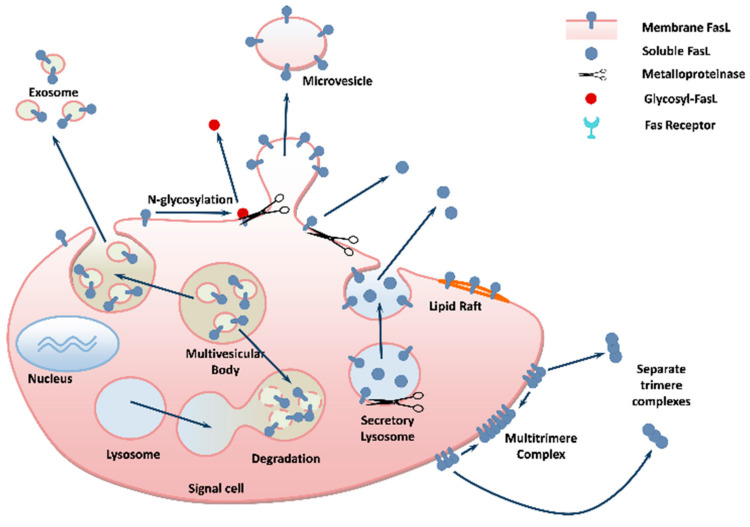
Mechanisms of FasL secretion by cells. Full-length FasL, termed membrane FasL (mFasL), is found in the cytosol, membrane bodies such as secretory lysosomes and multivesicular bodies, as well as on the cell surface. It may be released in full-length form embedded in the membrane of extracellular bodies as exosomes and microvesicles or, in a shorter version, cleaved by metalloproteinases and shed, termed soluble FasL (sFasL).

Oxidative stress is a known contributing aspect in physiological aging [26], which results from accumulation of oxidants generated during life in normal metabolism, in physiological inflammation, and also in various pathological states [27,28,29,30]. T-lymphocytes, microglial cells, endothelial cells and intestinal and lung epithelial cells are known to increase oxidative stress and induce FasL expression [28,31]. There is clear evidence that oxidative stress, in particular during aging, increases the number of extracellular vesicles that carry and release FasL protein [32]. Among them, we may find exosomes (small microvesicles 10–100 nm in diameter) [33], which are released from the multivesicular bodies through membrane invaginations [34] (see Figure 1) into the circulation and tissues [35]. Phosphorylation and ubiquitination of the FasL molecule are important for its transportation to secretory lysosomes [36]. Microvesicles containing FasL, on their surrounding membrane, were shown to negate T-cell immunity by induction of T-cell death (apoptosis) [37], and FasL, on exosomes, induced antigen-specific apoptosis in autologous CD4^+^ T-cells by a lymphoblastoid cell line [38]. This may be one of the reasons that exosomes have been indicated as potential biomarkers for aging-related diseases [39].

The literature reflects the involvement of reactive oxygen species (ROS) in FasL gene expression, both at the DNA [40,41] and the RNA [42,43] levels. In particular, the FasL promoter (FASLG), which is responsible for FasL DNA transcription, contains binding sites to transcription factors (e.g., NF-κB), known to be affected by a cell’s redox status [40,41]. Concomitantly, oxidative stress affects FasL mRNA [42,43] and microRNA-mediated FasL gene expression [44,45,46]. A short form (8 kDa) of FasL, which lacks a cytosolic transmembrane tail and a part of the extracellular domain, but retains two of the glycosylation sites of the extracellular domain [47], may result from alternative splicing of FasL mRNA.

Oxidative stress not only enhances FasL DNA and RNA with a subsequent increase in its protein levels; oxidative stress further affects the FasL protein itself. In particular, it promotes FasL cleavage via activation of metalloproteinases (MMPs) [48,49,50,51], which increase with age [26]. Many studies have reported increased cleavage and shedding of membrane FasL to its soluble form by MMPs directly from the cell’s membrane, and indirectly from secretory lysosomes which are further released from cells as extracellular vesicles [5,52,53,54] (see Figure 1). The details of the regulation of FasL cleavage and the generation of its soluble form are as yet incomplete. However, it is known that molecules such as 17β-estradiol upregulate both FasL transcription (increase RNA), and subsequent FasL cleavage by MMP3 (stromelysin-1) through estrogen receptor α (ERα) in osteoblasts and osteoclasts, and that MMP3 blockers inhibited sFasL production [55,56,57]. Similarly, T_H_2 cytokines, and IL-13 in particular, have been shown to increase shedding of FasL by increasing MMP7 activity in bronchial epithelial cells of patients with severe asthma [58]. This increase in shedding is accompanied by decreased FasL synthesis, and is presumed to be modulated through dephosphorylation of the proteins of the forkhead (FKHR) family, which facilitates their nuclear translocation and binding to the FKHR-responsive element of the FasL promoter [58], which may indicate regulatory feedback inhibition. 

In fact, the sFasL-mediated inhibition of apoptosis signaling vs. induction depends on the particular metalloproteinase that cleaves membrane FasL [53]. We have recently reported that MMP-7 knockout mice had decreased sFasL levels in their bloodstream, and further shown that these mice had attenuated lung fibrosis [59]. Particularly, fibroblasts with decreased expression of MMP-7, a shedder of sFasL, have been shown to increase sensitivity to apoptosis [59,60]. Concomitantly, high MMP7 decreases their sensitivity to apoptosis [54,61]. In a mirror situation, sFasL has been demonstrated to increase the production of ROS in neutrophils, with activation of inflammatory pathways [62]. In this context, oxidative stress is also a major factor, causing or exacerbating pulmonary diseases, including fibrosis [28,63,64,65].

## 4. Soluble FasL Increase in Serum of Patients with Pulmonary Disease

The pulmonary diseases associated with increased levels of the soluble form of FasL include (see Table 2): interstitial lung diseases (ILDs) such as hypersensitivity pneumonitis [66], idiopathic pulmonary fibrosis (IPF) [67], asbestosis and interstitial pneumonia [4,6,66,68]. Increased sFasL levels were also detected in patients diagnosed with lung cancer following chemotherapy, pulmonary sarcoidosis, pulmonary infections of varying etiology [69,70,71,72], acute lung injury (ALI) [73] and acute respiratory distress syndrome (ARDS) [54,73,74,75,76,77]. Similar changes were observed in fibrotic lung diseases in bronchoalveolar lavage fluid (BAL) [4,66]. In patients with chronic obstructive pulmonary disease (COPD), the levels of sFasL are directly related to the disease symptoms [78,79,80]. Patients with cachexic COPD show significantly increased serum sFasL levels compared to non-cachexic patients [78,79,80]. The sFasL content of serum is known to increase in patients diagnosed with asthma and is treated with omalizumab, and in allergic children during the symptomatic period [81,82]. Nevertheless, there is no general direction of change in sFasL serum levels that would be relevant for all asthma patients universally [83]; specifically, there is a significant decrease in plasma sFasL levels of patients with uncontrolled allergic asthma [83]. Of note, although increments in sFasL serum levels have been observed in numerous pulmonary pathologies, it was shown to decrease in pneumonic effusion, acute respiratory distress syndrome and lung cancer [4,6,54,68]. 

**Figure 2:**
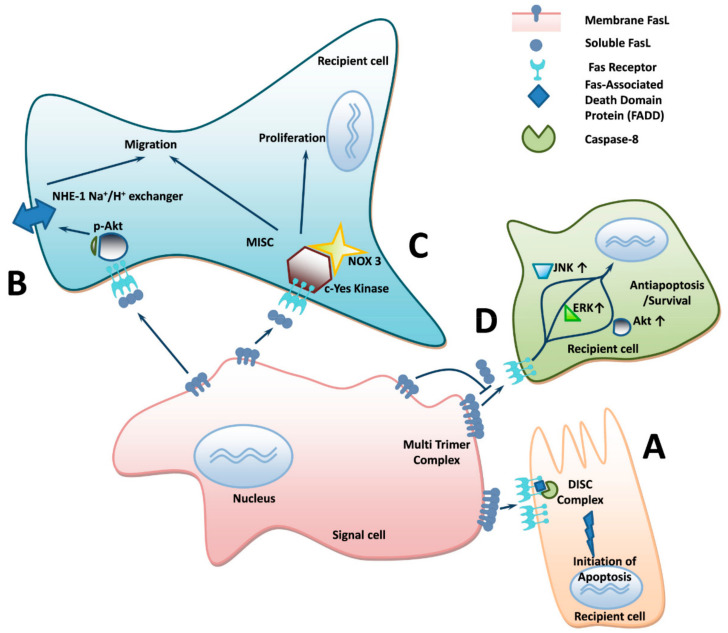
Mechanisms of FasL action on “target” cells. (**A**) Interaction of multiple trimers of membrane FasL with Fas receptors of recipient cells (cancer cells, cells with viral infection, etc.) initiates apoptosis. (**B**) Interaction of soluble form of FasL with Fas receptor, initiating cell migration in target cells. Cell motility is induced by PI3/Akt cascade, anchoring of cytoskeleton to NHE1, through interaction between C-terminal domain of the Na^+^/H^+^ exchanger and actin-binding proteins. (**C**) The motility-inducing signaling complex (MISC), Ca^2+^ influx, recruitment of NADPH oxidase-3 (NOX3) in MISC and c-Yes kinase activation. (**D**) sFasL competitive binding to Fas receptor produces anti-apoptotic and anti-cell death effects in nuclei of target cells via activation of pro-survival signaling cascades.

The specific roles of sFasL release in pulmonary diseases may vary per case. For instance, we [6] and others [68,86] have detected an anti-apoptotic role of sFasL (see Figure 2) as a mechanism of IPF by promoting immune suppression, immune privilege and immune escape [6,68,86,87,88,89]. We demonstrated that sFasL inhibits T-cell-induced apoptosis in IPF lung fibroblasts [6], in contrast to the role of mFasL in the cytotoxicity of CD4^+^ T-cells, which ensures their escape from immune surveillance [7], survival and proliferation in vitro, and in vivo in the experimental lung fibrosis and air pouch models [6]. Concomitantly, in other systems sFasL was demonstrated to promote cell survival as opposed to apoptosis by initiation of signaling pathways via JNK, Akt or ERK activation [9] (see Figure 2). For instance, sFasL binding to the Fas receptor of cell line GM6112 was shown to augment ERK1/2 activation, but not p38 [90]. sFasL induces proliferation in fibroblast-like synuviocytes from rheumatoid arthritis patients by activation of PI3K and caspase-8 signaling pathways, in addition to ERK signaling [91]. Although in the minority, it should be mentioned that a proapoptotic role has also been attributed to sFasL in lung pathologies such as ALI, ARDS and pulmonary adenocarcinoma [73,85,92]. This is akin to the role of membrane-bound Fas ligand [2,3,93]. 

Interaction of the soluble form of FasL with Fas receptor may also initiate cell migration in target cells (see Figure 2). This FasL-mediated cell migration is postulated to be involved in both the malignant transformation and the fibrotic processes [94]. All this allows not only cell survival, immune regulation and escape from immune surveillance, but also locomotion (e.g., cancer, fibrosis) with consequent disease progression.

## 5. Immune Disorders and Viral Infection Increase the Levels of Soluble FasL in Blood

Both soluble and membrane forms of FasL are known regulators of the immune system [9]. Soluble FasL is known to be involved in inflammation [2], including chronic inflammation [95] (see Table 3). Its serum concentration is elevated in patients with autoimmune lymphoproliferative syndrome [96]. Mice, exclusively expressing the soluble form of FasL, develop lymphadenopathy due to lack of selective immune cell-induced apoptosis usually performed by the membrane form of Fas ligand [97].

Soluble FasL levels have been consistently demonstrated to reflect the immune mobilization during viral and bacterial infection [75,86,101]. For instance, the concentrations of sFasL in serum and bronchoalveolar lavage (BAL) of asymptomatic carriers of human T-lymphotropic virus type-1 (HTLV-1) were associated with the percentage of CD4^+^ lymphocytes in BAL [86].

Serum sFasL supplementation may help prevent damage to glandular organs in Sjögren’s syndrome, which was demonstrated to be associated with decreased levels of sFasL in blood [99]. On the other hand, ex vivo depletion of sFasL was efficient in depleting alloreacting human donor anti-host T-cells in graft-versus-host disease [100]. 

Fas/mFasL signaling has only minimal involvement in both the mechanism of canine coronavirus type II-induced apoptosis [102] and SARS coronavirus-infected human dendritic cells [103], paving the way for the involvement of sFasL. Similarly, cell death induced in human dendritic cells by human coronavirus 229E (responsible for the common cold) was shown to be independent of FasL regulation [104].

## 6. Discussion

The distinction between the elevation of sFasL levels in the lung caused by disease, versus that caused by aging or even by immune disorders, including viral infection, is a challenging issue. It may be that that they are interconnected by nature. Interestingly, a linear correlation between age and the cubic root of serum sFasL concentration in humans was determined [18]. Sexual dimorphism adds additional complexity to this issue, considering that some pathologies may be overrepresented in one sex. Studies on women undergoing hormone therapy have detected increased serum sFasL levels as well [46]. 

Oxidative stress, and ROS in particular, is a known factor accompanying normal aging, as well as aging-associated diseases [63]. ROS contribute to cell senescence in the normal process of ontogenesis [63]. SASP is an important indication of cellular senescence, which is associated with lung pathologies [13]. As the role of FasL in the induction of pulmonary fibrosis has also been demonstrated in animals [6], the perspective of interconnection between soluble FasL and SASP presents itself as an interesting topic for further research. Fas ligand, and specifically its soluble form, is gaining recognition as an important factor involved in the progression of various pathological conditions [105].

The knowledge on sFasL involvement in viral diseases is still incomplete. Generally, sFasL release in viral infection is considered a part of the inflammatory immune response, specifically, as a potent chemo-attractant of human polymorphonuclear neutrophils [106]. In relation to lung diseases, serum or BAL sFasL was associated with fibrotic and inflammatory diseases [4,6,68,78] and cancer, including lung cancer [69,77,101]. 

sFasL has been suggested as a biomarker of disease progression in IPF [4,66], pneumonia [66,107], bronchiolitis obliterans [67], oxidative stress [63], cancer and aging [21]. There is a substantial translational potential for our understanding of the roles and regulation of various forms of released Fas ligand. This includes development of novel approaches to treatment with, for example, FasL-fused humanized antibodies to sensitize target cells to cell death [108,109,110] as suggested in glaucoma treatment [111,112], novel forms of RNA therapy [113], prognosis of long-term allergic outcomes at birth [114] or even for schizophrenia treatment [115]. However, there remain a number of outstanding problems that need to be addressed with regard to soluble Fas ligand as a therapeutic target. The switch between induction of apoptosis and other FasL-dependent signaling cascades, and the origin of the different FasL-associated signaling types through the Fas receptor, stand out as particularly important unanswered questions. Nevertheless, there is little doubt that FasL regulation will remain an important field of investigation in future biochemical and clinical studies. 

## Figures and Tables

**Table 1:** Changes in soluble Fas ligand (sFasL) levels during aging, and aged-related conditions and/or diseases.

Condition	Direction of Change	Notes	References
**Aging**			
Normal aging	↑	Serum	[17,18]
Aging-associated chronic inflammation	↓	Serum	[20,21]
Werner syndrome	↑	Serum	[17]
Age-related macular degeneration	↑	Plasma	[18]
Aging-associated T-cell population decrease	↑	Serum	[19]
Aging (athletes)	↓	Serum	[22]
Glaucoma	↓	Serum. Decreased ratio of sFasL to membrane FasL (mFasL) leads to increased susceptibility to fibrosis	[5]
**Oxidative Stress**			
**Condition**	**Direction of Change**	**Notes**	**References**
Disruption of spermatogenesis	↑	Testicular tissue	[24]
Renal degeneration	↑	Plasma, urine	[25]

**Table 2:** Changes in sFasL levels in serum and bronchoalveolar lavage fluid (BAL) of patients with lung disease.

Condition	Direction of Change	Notes	References
Idiopathic pulmonary fibrosis (IPF)	↑	Serum and BAL	[67,84]
Hypersensitivity pneumonitis	↑	Serum and BAL	[66]
Interstitial pneumonia	↑	Serum and BAL	[67]
Chronic obstructive pulmonary disease (COPD)	↮	Serum	[78]
Asthma	↮	Serum	[83]
Asthma (uncontrolled allergic patients)	↓	Serum	[83]
Asthma (omalizumab treatment)	↑	Serum	[81]
Asthma (allergic children)	↑	Serum, during symptomatic period	[82]
Acute lung injury	↑		[73]
Acute respiratory distress syndrome	↑	BAL of patients at risk of death	[85]
Lung cancer chemotherapy	↑	Serum	[77]
Lung cancer	↮	BAL	[69]
Small cell lung cancer	↑	Serum	[70]
Non-small cell lung cancer	↑	Serum	[71]
Pulmonary sarcoidosis	↑	BAL	[72]

**Table 3:** Changes in sFasL levels in immune-associated disorders.

Condition	Direction of Change	Notes	References
Inflammation	↑	Serum	[2]
Autoimmune lymphoproliferative syndrome	↑	Serum	[96,98]
Sjögren’s syndrome	↓	Serum	[99]
Chronic inflammation (pemphigus)	↑	Serum	[95]
Graft-versus-host disease	↑	Serum	[100]
Tuberculosis pleurisy	↑	BAL	[75]
Asymptomatic carriers of human T-lymphotropic virus type-1 (HTLV-1)	↑	Serum and BAL	[86]
